# It’s not just about recruitment: An exploratory look at tobacco education sessions to increase participation into smoking cessation programs among American Indians

**DOI:** 10.15761/hec.1000137

**Published:** 2018-05-14

**Authors:** CY Lumpkins, MK Filippi, N Nazir, CM Pacheco, CM Hester, SM Daley, WS Choi, CM Daley

**Affiliations:** 1 Department of Family Medicine, University of Kansas Medical Center, Kansas City, KS, 66160, USA; 2 Center for American Indian Community Health, University of Kansas Medical Center, Kansas City, KS, 66160, USA; 3 Department of Preventive Medicine & Public Health, University of Kansas Medical Center, Kansas City, KS, 66160, USA; 4 Center for American Indian Studies, Johnson County Community College, Kansas City, KS, 66210, USA; 5 United Health Care, Lawrence, KS; 6 National Hispanic Council on Aging

**Keywords:** american indian, native american, smoking cessation, recruitment, health education

## Abstract

American Indians (AI) have the highest smoking rates and lowest quit rates of any racial/ethnic group in the U.S. Researchers and community members from the American Indian Health Research and Education Alliance (AIHREA) created and evaluated a culturally-tailored smoking cessation program, All Nations Breath of Life (ANBL) as a recruitment tool for smoking cessation programs among AI. To increase enrollment in ANBL, AI smokers were approached at cultural events and asked to attend a 30-minute educational session (in-person, n= 179; tele-video, n=97). Tele-video (30%) and in-person (9%) session participants were recruited into ANBL. Pre- and post-tests showed participants in both sessions demonstrated increased motivation and confidence to quit smoking but significant differences were present in both sessions (p < 0.0001). Results indicate that theoretically guided and culturally tailored education sessions are viable approaches to educate and recruit underserved populations into programs that promote smoking cessation.

## Introduction

Commercial tobacco usage rates among American Indians (AI) are the highest when compared to all other racial and ethnic groups in the U.S. [1]. AI have the highest smoking rates (31.4%), followed most closely by whites (21.0%) and blacks (20.6%) [1]. In addition, AI demonstrate lower quit ratios (43%) than all other racial/ethnic groups (65%) [2]. Participation in smoking cessation and other health research programs is minimal among AI due to mistrust of health care providers, lack of knowledge concerning quit aids (i.e., pharmacotherapies), poor financial resources to purchase cessation treatment, inappropriate cultural messaging, and the failure to differentiate between traditional and recreational use [3]. While there is established research that shows the deepening impact of cultural barriers to participation in biomedical and public health research among underrepresented minority populations, little is known regarding recruitment and barriers that exist to prevent AI from participating in smoking cessation studies. In fact, few culturally tailored smoking cessation programs for AI exist (e.g. “Second Wind (Tainpeah, BlueEye),” [4], and fewer have been tested for efficacy and sustainability [5].

To better understand how to bolster AI participation into smoking cessation programs, we explored the utility of culturally tailored educational sessions as a recruitment tool. Theoretically guided and culturally tailored tobacco education sessions delivered by AI research staff provided participating AI communities with health information about the consequences of cigarette smoking. The team subsequently used the tobacco education sessions to recruit participants based on community engagement principles and Health Belief Model (HBM) constructs. Information gathered through this process provided a framework to influence and motivate individuals to quit smoking through a culturally appropriate and relevant program.

## Methods

### Recruitment

From August 2011 to November 2012 American Indian Health Research Education Alliance (AIHREA) team members recruited AI smokers, 18 years of age or older (N=276) for participation in tobacco education sessions (All Nations Breath of Life) [6] (N=24). Recruitment took place at various cultural and community events throughout the states of Kansas and Missouri. Most of the education sessions occurred during scheduled cultural and community events, (e.g., powwows and “Culture Nights”) and at a local urban AI center; other sessions took place at a tribal college campus. AIHREA obtained permission from the University of Kansas Medical Center Institutional Review Board to conduct the study and also from relevant review boards of community organizations to present the sessions.

Recruitment for the in-person education sessions was conducted on-site by AI team members through announcements at event venues, word of mouth, and personal invitation. These methods yielded 179 participants for 12 in-person sessions. Recruitment for the tele-video sessions was conducted in a similar fashion but incorporated the use of AI specific listservs and flyers and yielded 97 participants for the 12 tele-video sessions.

### Education sessions

An AI team member led the sessions (n=24) which lasted for 30 minutes. Prior to the start of each session, participants answered demographic and health-related questions, as well as a 12- question pre-test about their recreational smoking health knowledge. At the end of the session, participants took a 10-question post-test that mirrored the pre-test (Table 1). Participants also had the opportunity to sign up for All Nations Breath of Life (ANBL) but were not required to do so. Each participant received a $10 gift card for his/her time and participation.

The education sessions were interactive; participants were encouraged to ask questions and to make comments throughout the presentation. The educational material was delivered orally, a format preferred by AI participants in previous research conducted by the team. Each presenter was given a detailed outline of information to cover and then follow during all sessions. Given that the sessions were interactive and participants could ask questions and make comments, there were some differences among the discussions; however, the educational material was presented uniformly across sessions.

### Theoretical guide for education sessions

AI members of the research team conducted tobacco education sessions throughout regional AI community and cultural events. Throughout the sessions the team relayed information about health consequences of cigarette smoking, environmental tobacco smoke, and third hand smoke. The facts provided were specific and culturally tailored to AI populations and guided by components of the Health Belief Model [7]. HBM constructs are often used to explain or predict health behavior of an individual by pinpointing how an individual perceives a certain behavior; this may serve as a cue to help increase health promoting behavior to reduce risk [8]. Here, the research team prepared health promotion materials tailored to AI that were specifically designed to impact health and also recruitment behavior through education sessions. Content was designed to: 1) raise participants’ perceived susceptibility, 2) inform participants of the severity of potential outcomes related to smoking, 3) present participants with benefits of quitting smoking, 4) address barriers to quitting, 5) give “cues to action” (both with recommendations of actions the participants could take on their own and provide participants the opportunity to participate in ANBL), and 6) provide training and guidance to increase participants’ self-efficacy and confidence that they could quit should they decide to do so.

## Results

The average age of participants for the in-person session was 32 compared to 41 for tele-video sessions (*p* < 0.01). Participants attending the in-person sessions had more significant partners/spouses who “currently smoke” (31% versus 14%; *p* < 0.01). Participants in the in-person sessions smoked less days in the prior 30 days (19% versus 23%; *p*=0.02) and had been smoking for less years at their current rate (7% versus 11%; *p* < 0.01) than participants in the tele-video sessions. Participants in the in-person sessions also had lower dependence on nicotine, measured by the Fagerstrom Test for Nicotine Dependence (FTND, 2.77 versus 3.43; *p*=0.03), but were more likely to use other forms of tobacco (84% versus 15%; *p* < 0.01). Participants in both groups were similar across other variables (Table 2).

Pre- and post-test data for motivation and confidence to quit, and tobacco knowledge, were collected during both the in-person and tele-video sessions. Similar results were found in both arms. Participants who attended either session demonstrated an increased motivation to quit smoking; this increase was significant among in-person session participants *(p* < 0.01). Further, participants who attended either session demonstrated significant increased confidence to quit smoking (*p*=0.04 for in-person participants and *p* < 0.01 for tele-video participants). Knowledge scores of tobacco use and side effects of tobacco usage significantly increased from pre- to post-tests in both in-person (*p* < 0.01) and tele-video (*p* < 0.01) sessions.

Significant differences were found in total knowledge gains pre-test to post-test for in-person participants compared to tele-video participants (mean=1.27, SD=2.52; *p* < 0.01), but not for motivation or confidence. Similarly, overall pre- and post-test score comparisons were significant, including motivation to quit smoking (mean=0.89, SD=3.32; *p* < 0.01), confidence to quit smoking (mean=0.52, SD=2.64; *p* < 0.01), and knowledge (mean=3.58, SD=2.59; *p* < 0.01).

We compared session types to determine if participants entered into a culturally tailored quit smoking program (ANBL) after attending one of the education sessions. More than 9 % of the participants who attended an in-person session and 30% of the participants who attended a tele-video session were recruited into ANBL, even though all participants entered the sessions not planning to enroll in a quit smoking program.

## Discussion

Results suggest that education sessions theoretically guided by the HBM and community engagement principles were an effective way to inform AI smokers about smoking-related health outcomes and other tobacco facts specific to AI. This strategy also shows promise as a successful recruitment tool for AI programs similar to the All Nations Breath of Life (ANBL) smoking cessation program in the Midwest. While participants in both sessions experienced an increase in knowledge, the in-person sessions showed a greater impact on improved total knowledge. There was a significant increase in motivation and confidence to quit smoking for both sessions. Here, theoretically guided education sessions helped increase motivation and confidence to quit smoking among AI in this sample. This suggests that education sessions based on CBPR principles and guided by constructs of the HBM may be tools to use in recruitment and tailoring specific information. There is utility in applying theoretically driven health education that accounts for individual perceptions and beliefs. This approach may prove beneficial in demystifying interventions and health promotion programs targeted to minority, underserved and vulnerable populations. Here too, CBPR principles where members of AI were involved helped to strengthen recruitment efforts into smoking cessation programs.

In this study, the team was able to compare in-person versus tele-video tobacco education sessions to determine which was more successful at 1) improving participants’ tobacco knowledge and motivation to quit smoking, and 2) enrolling participants into the ANBL culturally tailored smoking cessation program. Present findings indicate that age, smoking status of significant partner/spouse, number of children living with a participant, number of days cigarettes were smoked in the past 30 days, years smoked at current smoking rate, nicotine dependence, and recreational use of other tobacco products may be important correlates of whether an individual participated in an in-person or tele-video tobacco education session. It is also possible that recruitment methods or locations for each type of session determined the type of individual to participate in that type of session.

Participants from both types of education sessions were recruited into the ANBL smoking cessation program (in-person, 9%; tele-video, 30%). AI smokers historically have lower quit rates and are less likely to enroll in smoking cessation programs than members of other racial/ethnic groups. Fu and colleagues [9] report the effectiveness of first identifying and then addressing specific factors that may impede or facilitate smoking cessation among AI. This process strengthens the potential for well-built interventions and also bolsters evaluation of smoking cessation programs. The ANBL program for this study incorporated similar strategies to promulgate healthy, sustainable and holistic lifestyles. This approach also addressed the desires of AI for smoking cessation programs and is consistent with the underlying notion that modifying recruitment messages based on community/participant perceptions may lead to an increase in enrollment for smoking cessation programs [10].

Other studies have reported factors that influence the success of study recruitment and retention, such as building rapport [11] increasing time commitment toward the study including cultural competence into creation of programs among others. Some, though not all participants, were familiar with research team members who conducted the education sessions. Many more participants knew of AIHREA and the goal to improve health in AI communities. In addition, the study used CBPR throughout, including community members in all aspects of the program. In fact, the ANBL smoking cessation program was developed at the request of many AI community members [12]. The education sessions were based on culturally appropriate messaging by stressing AI health as compared to generic themes of smoking as a health threat [13].

There were some limitations with the study that included sample selection process and managing recruitment of individuals into education sessions near the closing of the ANBL program. All participants included in the study self-identified as smokers however it is possible that not all participants were smokers. Participants also were recruited for the sessions (in-person vs. tele-video) by team members and were not randomized into sessions. In addition, the participants were recruited throughout the region and thus there may be variation in other AI communities where geographical, historical, and sociocultural characteristics differ. The sample did represent, however, a heterogeneous mix of tribes and localities (i.e., urban, rural, and reservation) and team members conducted purposive recruitment among AI community members to achieve a representative number in both in-person and tele-video sessions. Finally, recruitment for the ANBL effectiveness trial ended before the completion of the planned education sessions. The team chose to finish the scheduled education sessions although recruitment for the ANBL program had ended and offered these individuals an opportunity to participate in future programs. Therefore, it is possible that additional participants could have been recruited into the program. Future studies are needed to further test tobacco education sessions as a recruitment tool and the impact on health behavior [14]. Gathering additional empirical data among this population and others will be necessary to assess the effect of these sessions and whether all constructs within the HBM explain and predict eventual recruitment into smoking cessation programs. This study was a beginning to understanding the value of education sessions as a recruitment tool.

A major strength of this study was the use of education sessions (both in-person and tele-video) in recruiting for a culturally tailored quit smoking program. This study explored the impact of education sessions on motivation, confidence, knowledge and the utility in recruiting AI into a smoking cessation program. Pinpointing these individual-belief factors among AI members increases the potential to identify motivators and facilitators for smoking cessation behavior and also cues to action. These factors also may help health promoters to innovatively shape education via recruitment processes that lead to increased beneficial health promotion programming among AI populations.

## Figures and Tables

**Table 1.** Pre- and post-tests questions.

Number	Question
1	“On a scale of 0 – 10, with 0 being not at all important and 10 being very important, how important is it for you to quit smoking?”
2	“On a scale of 0 – 10, with 0 being not at all confident and 10 being very confident, assuming you decided to quit smoking, how confident are you that you could succeed?”
3	“At what age do American Indian children usually start smoking?” *Response categories allowed the participant to select:* “5 years old, 9 years old, 12 years old, or 15 years old”.
4	“American Indians have the highest rates of tobacco abuse of any ethnic group in the US.” *Responses categories were* “True or False”.
5	“How many chemicals that can cause cancer are found in cigarettes?” *Response categories included the options:* “100, 500, 1500, or4000”.
6	“If you smoked one pack of cigarettes per day, how much money could you save in a year by quitting?” *Response options were* “$500, $1000, $1500, or $2000”.
7	“Smoking can cause impotence in men.” *Response categories were* “True or False”.
8	“What is third hand smoke?” *Response categories included:* “A myth—it does not exist, Smoke from another person smoking in the same room as you, Smoke from another person smoking in the next room, Smoke left on clothing or furniture from someone previously smoking in the room”.
9	“Which of the following health conditions is NOT linked to smoking?” *Response options were* “Diabetes, Lung cancer, Heart disease, or High blood pressure”.
10	“How long does it take after you quit smoking to reduce your risk of sudden heart attack?” *Response categories were* “24 hours, one week, one month, or one year”.
11	“On average, smokers die ____ years before non-smokers.” *Response options were* “5, 8, 14, or 20”.
12	“Which of the following cancer is smoking linked to?” *Response categories were* “bladder, kidney, neither, or both”.

**Table 2.** Participant characteristics and bivariate analysis of session type among American Indian smokers.

Variable	In-person sessions (n=179)	Televideo sessions (n=97)	*p*-value*

Age; Mean (SD)	31.96 (14.02)	41.15 (17.79)	**< 0.01**

Gender n (%)			0.35
Male	90 (50.28)	43 (44.33)	
Female	89 (49.72)	54 (55.67)	

Education			0.22
Some high school	28 (15.64)	9 (9.38)	
High school/GED graduate	49 (27.37)	38 (39.58)	
Some college or tech school	74 (41.34)	33 (34.38)	
College graduate	24 (13.41)	13 (13.54)	
Graduate or professional school	4 (2.23)	3 (3.13)	

Smoking status of significant partner/spouse			**< 0.01**
Has never smoked	20 (11.24)	11 (11.34)	
Is a former smoker	20 (11.24)	9 (9.28)	
Current smoker	56 (31.46)	14 (14.43)	
No significant partner/spouse	82 (46.07)	63 (64.95)	

Number of children living with you; Mean (SD)	1.40 (1.59)	1.01 (1.48)	**0.04**

Age of smoking onset; Mean (SD)	15.61 (3.00)	16.16 (3.00)	0.17

How many of the past 30 days did you smoke cigarettes n (%)	19.49 (12.37)	23.11 (11.19)	**0.02**

How many cigarettes smoked per day n (%)	8.38 (7.41)	9.77 (7.48)	0.17

How long have you smoked at your current rate? (years)	7.61 (6.95)	11.77 (8.86)	**< 0.01**

FTND* where 0=low dependence, 10=high dependence; Mean (SD)	2.77 (2.42)	3.43 (2.24)	**0.03**

Other recreational tobacco use			
Yes	49 (28.99)	9 (10.11)	**< 0.01**
No	120 (71.01)	80 (89.89)	

* FTND = Fagerstrom Test for Nicotine Dependence

* *p*-value is based on the chi-square test
